# *HSAT3* lncRNA regulates replication re-initiation and colocalizes with TOP2A under stress conditions

**DOI:** 10.1186/s13072-026-00707-9

**Published:** 2026-10-24

**Authors:** Julian Kanne, Felix Heß, Wei Min, Jörg Isensee, Elena Wasserburger-Zichel, Nikolai Tschernoster, Luca Dartenne, Sascha Hoppe, Janine Altmüller, Margarete Odenthal, Tim Hucho, Christina Grimm, Michal R. Schweiger

**Affiliations:** 1 Institute for Translational Epigenetics, University of Cologne, Faculty of Medicine and University Hospital Cologne, 50931 Cologne, Germany; 2 Center for Molecular Medicine Cologne, University of Cologne, Faculty of Medicine and University Hospital Cologne, 50931 Cologne, Germany; 3 Department of Anesthesiology and Intensive Care Medicine, Translational Pain Research, University of Cologne, Faculty of Medicine and University Hospital Cologne, 50931 Cologne, Germany; 4 Cologne Center for Genomics (CCG), University of Cologne, 50931 Cologne, Germany; 5 Present Address: Max-Delbrück Center for Molecular Medicine, Genomics, 10115 Berlin, Germany; 6 Institute of Pathology, University of Cologne, Faculty of Medicine and University Hospital Cologne, 50937 Cologne, Germany; 7 Center of Integrated Oncology Cologne-Bonn, University of Cologne, Faculty of Medicine and University Hospital Cologne, 50937 Cologne, Germany; 8 West German Genome Center (WGGC), Cologne Site, University of Cologne, 50937, Cologne, Germany

**Keywords:** Long noncoding RNA, Satellite 3, Satellite III, *HSATIII*, *SATIII*, *SAT3*

## Abstract

**Background:**

Long non-coding RNAs transcribed from pericentromeric Satellite 3 repeats (*HSAT3*) are involved in stress-dependent splicing processes and maintenance of genome stability. The functions of the transcripts are largely unknown, which is mainly due to the predominant heterochromatic organization of satellite regions. *HSAT3* expression is induced upon several cellular stressors and builds nuclear stress bodies (nSB), which recruit a large number of RNA-binding and regulatory proteins. Recently, we reported that *HSAT3* transcripts induce resistance against the topoisomerase 2a (TOP2A) inhibitor etoposide in lung cancer.

**Results:**

We show that *HSAT3* transcripts and topoisomerase IIα (TOP2A) accumulate in nuclear stress bodies. Biochemical assays indicate that *HSAT3* RNA can associate with TOP2A. Using single-molecule DNA fiber analysis, we demonstrate that depletion of *HSAT3* delays replication re-initiation. Consistent with this, *HSAT3* knockdown alters cell cycle distribution in the presence of the TOP2A inhibitor etoposide. *In vivo*, reduced *HSAT3* levels lead to delayed tumor growth in xenograft models. In addition, we identify epigenetic compounds that suppress *HSAT3* expression and enhance cellular sensitivity to etoposide, suggesting a potential strategy for combination treatments.

**Conclusions:**

Together, our findings support a functional link between stress-induced repetitive RNAs, nuclear compartmentalization, and replication-associated processes. These results provide a framework for understanding how pericentromeric lncRNAs contribute to genome stability and drug response in cancer cells.

**Supplementary Information:**

The online version contains supplementary material available at https://doi.org/10.1186/s13072-026-00707-9.

## Background

Long non-coding RNAs (lncRNAs) play important roles in epigenetic regulation and are increasingly implicated in cancer development and progression [1]. Among these, repetitive satellite sequences located in centromeric and pericentromeric regions have traditionally been considered structural elements that contribute to chromosomal stability and proper mitotic progression [2–5]. Under homeostatic conditions, the majority of pericentromeric regions are maintained in a heterochromatic state, characterized by high levels of DNA methylation, repressive histone modifications such as H3K9me2/3 and H4K20me3, and the presence of heterochromatin protein 1 (HP1) [6–8].

Upon cellular stress, this repressive state can be partially relaxed, leading to the transcriptional activation of satellite-derived non-coding RNAs. A prominent example is the primate-specific Satellite 3 RNA (*HSAT3*), which is already expressed at basal levels under normal conditions and highly induced in response to various stressors, including thermal stress, chemotherapeutic treatment, proteotoxic stress, and heavy metal exposure [9, 10].

Upon stress *HSAT3* transcripts accumulate in distinct nuclear compartments termed nuclear stress bodies (nSBs), which form in close proximity to their genomic loci, most prominently at the 9q12 region [9–13]. These membrane-less structures recruit a wide range of RNA-binding proteins and splicing regulators and have been linked to stress-induced changes in RNA processing, including widespread intron retention during recovery from heat stress [14–16]. Mechanistically, *HSAT3* contributes to these processes by recruiting factors such as CLK1 and YTHDC1, thereby modulating phosphorylation-dependent and m6A-dependent splicing pathways [3, 15, 16].

The expression of *HSAT3* is primarily regulated by heat shock factor 1 (HSF1) and bromodomain and extraterminal domain (BET) proteins, including BRD4 [17–20]. Both pathways are frequently dysregulated in cancer, and elevated *HSAT3* expression has been reported in multiple tumor types [21–23]. In addition to its role in transcriptional regulation, BRD4 has been implicated in the control of RNA processing during cellular stress. Notably, BRD4 regulates alternative splicing during heat shock and contributes to stress-induced splicing changes [18]. Consistently, BRD4 is also involved in the formation of *HSAT3*-containing nuclear stress bodies, and pharmacological inhibition of BET proteins suppresses *HSAT3* expression and nuclear stress body formation [19, 20]. Furthermore, perturbations of heterochromatin integrity, such as loss of BRCA1-dependent ubiquitination or altered histone modification patterns, can lead to aberrant activation of satellite transcription. This has been associated with genome instability, activation of DNA damage responses, and the formation of RNA hybrids (R-loops) [24–29].

Emerging evidence suggests that satellite RNAs can directly influence DNA replication. Overexpression of murine satellite transcripts has been shown to induce replication stress and impair replication fidelity [26], while proper replication of pericentromeric regions is essential for maintaining genome stability [28]. Proteins such as TRF2 and the helicase RTEL1 have been implicated in facilitating replication through these repetitive regions [30].

Recently, we demonstrated that increased *HSAT3* expression confers resistance to the topoisomerase IIα (TOP2A) inhibitor etoposide in lung cancer models [19]. TOP2A binds to dsDNA and introduces strand breaks to resolve topological problems that arise during transcription and replication [31]. However, the role of *HSAT3* in DNA replication dynamics and its mechanistic relationship with TOP2A remain largely unclear. In addition, given that *HSAT3* expression is tightly controlled by epigenetic regulators such as BET proteins, it is of particular interest whether pharmacological modulation of chromatin-associated pathways can influence *HSAT3* levels and thereby alter cellular responses to chemotherapeutic agents.

Here, we investigate the functional interplay between *HSAT3*, TOP2A, and DNA replication under stress conditions. Furthermore, we systematically explore epigenetic regulators of *HSAT3* expression using a focused compound screen and assess their potential to modulate replication-associated phenotypes and drug sensitivity. Together, our study provides evidence that *HSAT3* contributes to the regulation of replication re-initiation and influences cellular responses to chemotherapeutic treatment.

## Materials and methods

### Cell lines and heat stress conditions

HeLa cells (RRID: CVCL_0030) and A549 cells (RRID: CVCL_0023) were cultivated in Dulbecco’s Modified Eagle’s Medium (Biochrom), containing 10% fetal calf serum (Sigma-Aldrich), 2 mM L-glutamine (Sigma-Aldrich) and 100 U/ml penicillin/streptomycin (Sigma-Aldrich). The HCC827 cell line (RRID: CVCL_2063) was kindly provided by Prof. Dr. Roman Thomas, University of Cologne, Medical Faculty. HCC827 cells stably expressing an shRNA targeting *HSAT3* or GFP were generated by viral transduction and described previously [19]. Sequences for the integrated shRNAs are provided in Supplementary table S1. HCC827 was cultivated in RPMI 1640 medium, containing 10% fetal calf serum, 2 mM L-glutamine, and 100 U/ml penicillin/streptomycin. All cell lines were tested negative for mycoplasma using the Mycoalert Plus Mycoplasma detection kit (Lonza) according to the manufacturer’s protocol. For heat stress induction, cells were incubated at 44 °C for 1 h with 5% CO_2,_ 95% hmidity. Heat stress with recovery was carried out at 1 h 44 °C followed by a recovery phase at 37 °C, 5% CO_2_, 95% humidity for the indicated time.

### Transfection

Transfections were performed with the respective siRNAs (*HSAT3*, Control) using Lipofectamine RNAiMAX reagent (Invitrogen Inc., Cat. No. 13778030) according to the manufacturer’s recommendations. Additionally, a modified antisense oligonucleotide targeting *HSAT3* was transfected using Lipofectamine 2000 (Invitrogen Inc., #11668027). Sequences of siRNAs and antisense-oligos were taken from Kanne et al. [19] and are provided in Supplementary Table S1.

### Immunofluorescence followed by RNA fluorescence in situ hybridization (FISH)

TOP2A immunofluorescence, followed by *HSAT3* RNA-FISH, was performed as follows: Cells were fixed in 70% ethanol / 0.03% Triton X-100 for one hour at 4 °C, then blocked for 15 min with 0.5% BSA / 0.03% Triton X-100. The cells were then incubated overnight at 4 °C with a TOP2A antibody (Sigma-Aldrich, Cat. No. SAB4502998, RRID: AB_10747477), followed by incubation with a goat anti-rabbit IgG (H + L) secondary antibody coupled to Alexa Fluor^™^ 488 (Life Technologies, Cat. No. A11034, RRID: AB_2576217) for one hour at room temperature. The cells were then washed in Stellaris wash buffer A (BioSearch Technologies). Stellaris hybridization buffer (BioSearch Technologies), containing three *HSAT3* RNA-binding probes (25 nM each), was added, and the cells were incubated for four hours at 37 °C. Then the protocol for RNA fluorescence in situ hybridization was followed.

### RNA fluorescence in situ hybridization (FISH)

FISH experiments were performed as described before [19]. In brief, cells were fixed for 10 min with 1% formaldehyde (methanol-free) (Carl Roth Inc.) and permeabilized with 70% ethanol at 4 °C overnight. For subsequent RNA single molecule FISH (smFISH) stainings, cells were washed with Stellaris wash buffer A (BioSearch Technologies). Stellaris hybridization buffer (BioSearch Technologies) containing, three *HSAT3* RNA-binding probes labelled with Quasar 570 (25 nM each), was added. The cells were then incubated overnight at 37 °C. After washing with Stellaris wash buffer A, the nuclei were stained with Hoechst stain 33,342 (PromoKine, Cat. No. PK-CA707-4044). The cells were then washed with Stellaris wash buffer B for five minutes at room temperature. 96-well plates subjected to high-content screening were stored in PBS at 4 °C until analysed by microscopy (see below). For standard immunofluorescence, coverslips were mounted on glass slides using Fluoromount-G (eBioScience, Cat. No.00-4958-02) and images were obtained using a Keyence BZ-X810 fluorescence microscope with a 60× objective and a resolution of 960 × 720 pixels (~ 0.25 μm/pixel). Sequences of the *HSAT3* RNA-binding probe are provided in Supplementary Table 1.

### Quantitative high-content screening microscopy

Plates were scanned using a Thermo Fisher Cellomics ArrayScan VTI and images of 512 × 512 pixels were acquired with a 20x objective and analyzed using the Cellomics software package (Colocalization V.4 Bioapplication). Cell nuclei were identified by Hoechst staining and according to the object identification parameters size: 100-1,200 µm^2^, ratio of perimeter squared to 4π area: 1–2, length-to-width ratio: 1–2, average intensity: 50 − 1,000, total intensity: 3 × 10^4^-2 × 10^7^. Nuclear stress bodies were identified within a circular region extending the nucleus by maximally 20 μm. The object identification parameters for nSB were: 1.5–20 µm^2^, ratio of perimeter squared to 4 π area: 1-1.8, length-to-width ratio: 1-1.8, average intensity: 100–1500, total intensity: 5 × 10^3^-5 × 10^4^.

### Foci detection and colocalization analysis

Nuclei were segmented using StarDist 2D pre-trained model on DAPI channel [32]. TOP2A and *HSAT3* foci were detected by Foci Analyzer (v1.92) plugin, which is freely available at: https://github.com/BioImaging-NKI/Foci-analyzer. The parameters were consistently applied: marker-controlled watershed, Gaussian blur: average 2 pixels; min area: 5–7 pixels [0.31–0.44 μm², ~ 0.63–0.75 μm diameter]; max area: 120 pixels [~ 7.5 μm², ~ 3.1 μm diameter]; 0.25 μm/pixel calibration. Thresholds were optimized through previewing to match foci morphology, for TOP2A: 0.3–0.5 μm puncta [33]; *HSAT3*: 1–2 μm clusters [34]. The counts of TOP2A foci, *HSAT3* foci, colocalized foci, and Manders’ coefficients (M1, M2) were exported.

### Spectral unmixing

Bleed-through and cross-talk between the TOP2A and *HSAT3* channels were modified by using linear correction [35]. Single-stained controls, after 6-hour recovery period, yielded bleed-through factors of *f*1 = 0.098 (*HSAT3*→TOP2A) and *f*2 = 0.024 (TOP2A→*HSAT3*; additional details are available in the Supplementary Materials). The corrected intensities, based on the mean gray values obtained from Fiji, were calculated as follows: Intensity_green, corrected_ = Intensity_green, raw_ – (f1xIntensity_red, raw_) and Intensity_red, corrected_ = Intensity_red, raw_ – (f1xIntensity_green, raw_). The entire spectral unmixing process was performed in Fiji (v1.54p), followed by channel merging.

### Statistical analysis for colocalization analysis

44–120 nuclei were analyzed per experimental condition on three different images (total nuclei count = 456). For TOP2A, *HSAT3*, and colocalized foci counts, differences among groups were evaluated using one-way ANOVA followed by Tukey’s multiple comparison test, and data are presented as mean ± standard deviation (SD). For M1 and M2, which exhibited non-normal distributions, the Kruskal-Wallis test followed by Dunn’s multiple comparison test was applied, and data are expressed as median ± interquartile range (IQR). And all statistical analysis were conducted in GraphPad Prism 10.1.2. Statistically significant differences are indicated by asterisks (*adjusted *P* < 0.05, **adjusted *P* < 0.01, ***adjusted *P* < 0.001).

### In vitro RNA pulldown assay

The biotinylated *HSAT3* RNA oligo GGAAUGGAAUGGAAUGGAAU-TEG-Biotin and the negative control oligo Biotin-CCCCCAAAAAUUUUUGGGGG were purchased from biomers.net. Pulldown assays were performed as described previously with minor modifications [15]. In brief, Hela cells were lysed in 25 mM Tris-HCl pH 7,4, 150 mM NaCl, 1 mM EDTA, 1% NP-40, 5% Glycerin, 1x complete, Mini, EDTA-free Protease inhibitor cocktail (Sigma-Aldrich) and incubated with Dynabeads^TM^ M-280 Streptavidin (Thermo Fisher) for preclearing for 1 h at 4 °C by head-to-head rotation. The supernatant was collected and the precleared cell lysate (2 mg protein) in 100 µl was used in the in vitro pull down. 200 pmol of biotinylated RNA-oligo was bound to 20 µl Dynabeads^TM^ M-280 Streptavidin in 1xPBS, 0.1% Triton X-100, 0.6 mM Phenylmethylsulfonyl fluoride (PMSF) for 1 h at 4 °C, washed 3 times in 1xPBS, 0.1% Triton X-100, 0.6 mM PMSF and resuspended in 200 µl 1x PBS, 0.1% Triton X-100, 0.6 mM PMSF, 1x complete, Mini, EDTA-free Protease inhibitor cocktail and RNase inhibitor. Subsequently, 100 µl precleared cell lysate (2 mg protein) was added and incubated for 2 h at 4 °C with head-to-head rotation. After washing the Dynabeads five times with cold PBS containing 0.1% Triton X-100, the co-precipitated proteins were eluted from the Dynabeads in SDS sample loading buffer for 5 min at 95 °C and subjected to western blot analyses. Western blots were incubated with the primary antibodies: TOP2A (Sigma-Aldrich, Cat. No. HPA006458, RRID: AB_1858189) or ALYREF (Santa Cruz, Cat. No. sc32311, RRID: AB_626667),washed three times (10 min each with TBST-0.1%Tween-20) and subsequently incubated with peroxidase-conjugated secondary antibodies. Chemiluminescence was performed using Clarity Western ECL Substrate (BioRad) and images were taken with a ChemiDoc^TM^ MP Imaging System (Bio-Rad).

### DNA fiber assay

Experiments were performed following instructions by Mendez-Bermudez et al. [30] with minor modifications. 1 × 10^6^ HeLa cells were seeded in a 6-well plate and transfected 24 h later as described before. 48 h after transfection, cells were pulse-labeled for 30 min with 20 µM final concentration iododeoxyuridine, IdU (Sigma-Aldrich). Cells were washed once with PBS and cell cycle progression was then blocked by incubation with 1 mM thymidine (Sigma-Aldrich) for 1 h. Subsequently, cells were washed once with PBS and pulse-labeled for 30 min with 100 µM chlorodeoxyuridine, CldU (MP Biomedicals). Cells were collected and embedded in 2% low melting agarose plugs. DNA plugs were incubated for 48 h at 42 °C with Proteinase K (1% SDS, 0.25 M EDTA, pH 8.0 and 1 mg/ml Proteinase K). Next, agarose plugs were heated at 68 °C for 15 min in 1X TE containing β-Agarase I buffer (NEB). The melted agarose was cooled down to 42 °C and incubated for 48 h in the presence of β-Agarase (NEB) and 30 min with 0.25 M 2-(N-morpholino) ethanesulfonic acid, pH 5.5. DNA present in the melted β-Agarase-digested agarose was stretched onto silanized coverslips. DNA molecules were stretched at a constant speed (300 μm/sec) on silanized coverslips with an automated combing device (FiberComb, GENOMIC VISION). This procedure gives a uniform stretching factor of 2 kb/µm across the surface. Next, slides were denatured in 1 N NaOH for 8 min at room temperature, rinsed in 1X PBS at 4 °C for 5 min and dehydrated in increasing concentrations of ethanol for 5 min each (50%, 70%, 90% and 100%) at room temperature. The slides were then incubated with blocking solution (1.5% blocking reagent, Roche and 0.05% Tween 20) for 30 min at 37 °C followed by incubation with mouse anti-BrdU-B44 (BD Biosciences, Cat. No. 347580, RRID: AB_400326) targeting IdU, together with rat anti-BrdU Clone BU1/75 (Abcam, Cat. No. ab6326, RRID: AB_305426) antibody, targeting CldU for 30 min at 37 °C. Next, the samples were washed in NaCl 0.5 M, Tris 20 mM pH 7.8, Tween-20 0.5%, for 6 min at room temperature. Samples were then incubated for 30 min at 37 °C with secondary antibodies goat anti-mouse Cy3 (Abcam, Cat. No. ab6946, RRID: AB_955045) together with a goat anti-rat Cy5 (Abcam, Cat. No. ab6565, RRID: AB_955063). After washing in 1X PBS, the slides were again dehydrated in increasing concentrations of ethanol for 5 min each. Analyses of the slides were performed in FiberVision Automated Scanner. To calculate track lengths, only IdU tracks flanked by CldU tracks present in intact fibers, established by DNA counterstaining, were measured. To determine the length in kb/min, the ratio between the lengths of the IdU + CldU tracks divided by the labeling time in minutes was calculated using the FiberStudio 2.0.2 software (Genomic Vision). Replication re-initiation was calculated as the ratio of IdU/CldU. In our settings, CldU labeled the reinitiated DNA track, therefore higher values represent delayed re-initiation. Statistical analysis of the median distributions was calculated with the nonparametric Mann-Whitney rank sum test using GraphPad Prism 9 software (GraphPad Software, RRID: SCR_002798).

### Cell cycle analyses

To determine the cell cycle stage, the distribution of the total Hoechst intensity values acquired from quantitative high-content screening microscopy data were measured. Four thresholds were adjusted manually to gate cells in 2 N (G0/G1), 2–4 N (S), and 4 N (G2/M) stages, respectively. Gates were applied to evaluate the mean number of foci in cells of the respective cell cycle stage. Single cell data were further processed and plotted using custom R scripts.

### Cell proliferation assay

Cells were incubated at 37 °C with 1% DMSO and every 30 min two images were taken for 48 h by ImageXPress Micro4 (Molecular Devices). Images were analyzed using CellProfiler software (Broad Institute).

### Cell line-derived xenograft (CDX) models

10-week-old immunodeficient female mice (Rj: NMRI-Foxn1^nu/nu^) were obtained from Janvier labs and were imported into the animal facility of the pathology of the University Clinic Cologne and allowed to acclimatize for one week. Mice were housed under specific pathogen free conditions in individually ventilated cages with a 12/12 h light/dark cycle with rodent chow and water *ad libitum*. Four animals were housed per cage. The experiments were performed in accordance with the Federation of European Laboratory Animal Science Association (FELASA) recommendations, the German animal welfare law and the ARRIVE guidelines. The study was approved by the Landesamt für Natur, Umwelt und Verbraucherschutz (LANUV), Nordrhein Westfalen, Germany under the license number AZ 84-02.042015.A541. Mice were anaesthetized by inhalation of isoflurane and 2 × 10^7^ cells (HCC827-shGFP or HCC827-sh*HSAT3*) were subcutaneously injected into each flank of a mouse. Per group 21 tumors from 14 mice were analyzed. Tumor size was determined by calliper measurements and tumor volume was calculated using the formula length x width^2^ / 2. Experiments were performed blinded for the two groups.

### Immunohistochemistry

Xenograft tumors were dissected, fixed in 4% Formaldehyde/PBS and embedded in paraffine. 3 μm sections were deparaffinized and antigens retrived by heating for 20 min in citrate buffer (pH 6.0). Blocking was performed in 5% goat serum in PBS for 1 h at room temperature. Antibodies targeting cleaved caspase 3 (Cell Signaling, Cat. No. 9661, RRID: AB_2341188, dilution: 1:100) and Ki67 (abcam, Cat. No. ab15580, RRID: AB_443209, dilution 1:200) were incubated overnight at 4 °C. Sections were washed in PBS and incubated for 1 h with a biotinylated secondary antibody (anti-rabbit IgG, 1:500). Signals were detected using the Vectastain ABC kit with the substrate diaminobenzidine and sections were counterstained with hematoxylin.

### Compound screen library composition and performance

A customized epigenetic drug library of 178 compounds was obtained from TargetMol Corp. in cooperation with BioCat GmbH. Table S2 lists the compound library’s composition. Cells were plated in a 96 well format for the assays used: qPCR, HCS-FISH and cell viability test with AlamarBlue^®^ reagent (Life Technologies GmbH). Compounds were added in a random fashion, independent from structural and functional properties. To further sustain a high degree of comparability, cells were constantly seeded with a density of 7 × 10^3^ cells per well for the qPCR assay and 1 × 10^4^ cells per well for the RNA FISH. Preliminary experiments showed that 24 h of drug treatment were sufficient to generate prominent effects on *HSAT3* expression. Thus, heat shock (HS) for 1 h at 44 °C was applied 24 h after drug treatment and cells were harvested immediately after HS. Each plate contained three positive controls (JQ1) and three negative controls (DMSO 0.1%). Two replicates were performed for each drug. *HSAT3* expression was calculated as described in section “Two-step RNA Lysis - RT - qPCR assay used for compound screen” and *HSAT3* foci number was calculated as mean foci over all cells. The majority of compounds were applied at a concentration of 1 µM. The compound concentrations, the screen results and the numerical data can be found in Table S2.

### Quantitative PCR (qPCR)

qPCR analysis beyond the compound screen were performed as follows: the double-strand DNA-binding dye SYBR Green, which is included in the GoTaq qPCR Master Mix (Promega), was used to detect accumulating PCR fragments. 5 ng of reverse transcribed cDNA were mixed with 300 nM primers and 1x GoTaq qPCR Master Mix to a final reaction volume of 10 µl. Cycling parameters were used according to the manufacturer’s protocol. Sequences of qPCR primers are provided in Supplementary Table S1.

### Two-step RNA Lysis - RT - qPCR assay used for compound screen

For RNA extraction, ExCellenCT Lysis Kit (Applied Biological Materials Inc.) was used. The RNA lysate was used in a one-step protocol combining reverse transcription and qPCR in one PCR run. For this, Luna Universal One-Step RT-qPCR Kit (New England Biolabs) was employed. The protocol was performed following the manufacturer’s instructions including minor modifications.

Relative *HSAT3* expression levels were calculated by the comparative Ct (cycle of threshold) also known as ΔΔCt method. Ct-values of *HSAT3* were normalized to a corresponding endogenous control RNA (*Tubulin Beta Class I*, *TUBB*) and then compared to the solvent-treated samples (DMSO). Oligos are provided in Supplementary Table S1.

### Cell viability assay

Cell viability was measured using the AlamarBlue^®^ Cell Viability Assay (Life Technologies, Cat. No. DAL1025) according to the manufacturer’s recommendations. After transfection or drug treatments, 10 µL of AlamarBlue^®^ reagent was added to 100 µl medium in 96-well plates. Fluorescence intensities were measured 3 h after addition of the reagent by an Infinite 200 PRO Tecan Microplate Reader (Tecan Germany GmbH).

### Statistical analysis

Statistical analyses were performed using the GraphPad Prism (GraphPad Software, RRID: SCR_002798). For all tests, p value significance was defined as follows: not significant (n.s.) *p* > 0.05; *p < 0.05; **p < 0.01; ***p < 0.001; ****p < 0.0001.

## Results

### TOP2A is recruited to nuclear stress bodies during thermal stress recovery and associates with *HSAT3* RNA

We previously observed that TOP2A localizes to nuclear stress bodies (nSBs) following heat stress (1 h at 44 °C) and subsequent recovery (24 h at 37 °C), but not immediately after heat shock [19]. To further characterize the kinetics of this process, we performed a time-course analysis of TOP2A recruitment during recovery from thermal stress.

**Fig. 1:**
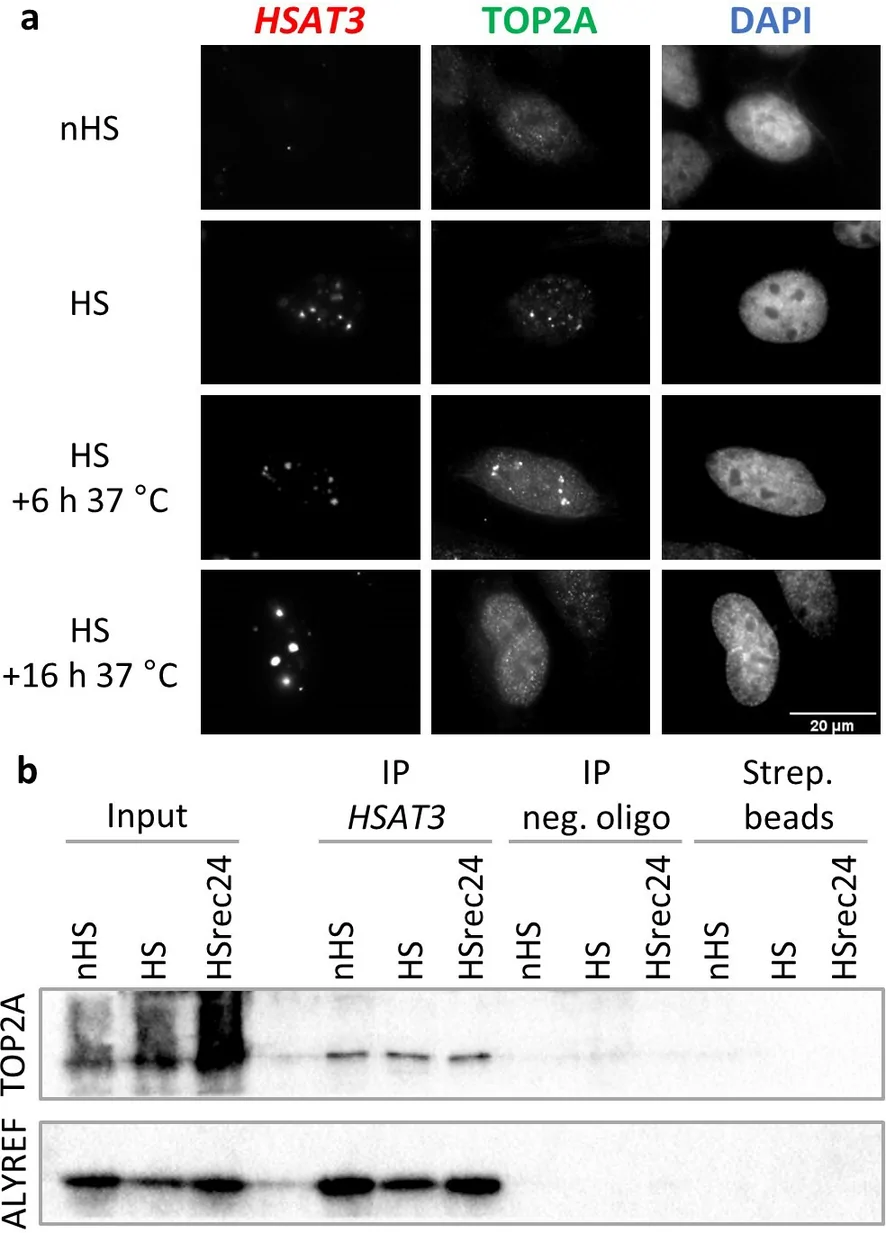
TOP2A is recruited to nSB during thermal stress recovery. **a** Localization of TOP2A to nSBs. HeLa cells were subjected to heat stress for 1 h at 44 °C (HS) followed by the indicated recovery times at 37 °C or left at 37 °C (nHS) TOP2A immunofluorescence with *HSAT3* RNA-FISH. Nuclei were stained with Hoechst 33,258. **b** In vitro pulldown assays of RNA-protein (RNP) complexes with *HSAT3*. A biotinylated *HSAT3*-RNA oligo (GGAAU)x4, a negative control oligo (CCCCCAAAAAUUUUUGGGGG) or streptavidine-magnetic beads were incubated with HeLa cell extracts. HeLa cells were kept at 37 °C (nHS), exposed to thermal stress for 1 h at 44 °C (HS) or followed by 24 h recovery at 37 °C (HSrec24). 12,5% of the Input (180 µg) was loaded on the SDS page. TOP2A and ALYREF were detected by Western blotting

HeLa cells were subjected to heat stress (1 h at 44 °C) followed by recovery periods ranging from 0 to 24 h at 37 °C (Fig. 1a, Additional Figs. 1 and 2) or kept at 37 °C (no heat stress, nHS) Under non-stress conditions, *HSAT3* and TOP2A foci were rarely detected. Immediately after heat stress, both *HSAT3* and TOP2A foci were induced and showed partial colocalization. This colocalization persisted for up to ~ 16 h during recovery, whereas at later time points (≥ 20 h), *HSAT3* foci became less frequent and TOP2A colocalization decreased (Additional Figs. 1, 2 and 3). Following heat stress (1 h at 42 °C), we also observed colocalization of *HSAT3* and TOP2A in the non-small cell lung cancer cell line A549 (Additional Fig. 4). Immunofluorescence analysis in HCC827 cells was precluded by detachment of the cells from the coverslips by the heat stress procedure.

Given this spatial association, we next asked whether TOP2A directly interacts with *HSAT3* RNA. We performed *in vitro *RNA pulldown assays using a biotinylated *HSAT3* RNA oligonucleotide consisting of GGAAU repeats, which represent a major component of *HSAT3* transcripts [2, 15, 20]. TOP2A was consistently detected in (GGAAU)x4 pulldown fractions from HeLa cell lysates under non-stress, heat stress, and recovery conditions (Fig. 1b), as well as in HCC827 and A549 cell lysates under the two tested conditions (non-stress and heat stress, Additional Fig. 5). This indicates that TOP2A can associate with *HSAT3* RNA sequences. The RNA-binding protein ALYREF served as a positive control and was efficiently co-precipitated, consistent with previous reports [15, 16, 20].

### *HSAT3* depletion alters cell cycle distribution under etoposide treatment

Heat stress induces transient cell cycle arrest at the G1/S and G2/M checkpoints [36], and nSB formation has been reported to occur predominantly in late S/early G2 phases [19]. We therefore investigated whether *HSAT3* depletion affects cell cycle progression following heat stress.

**Fig. 2:**
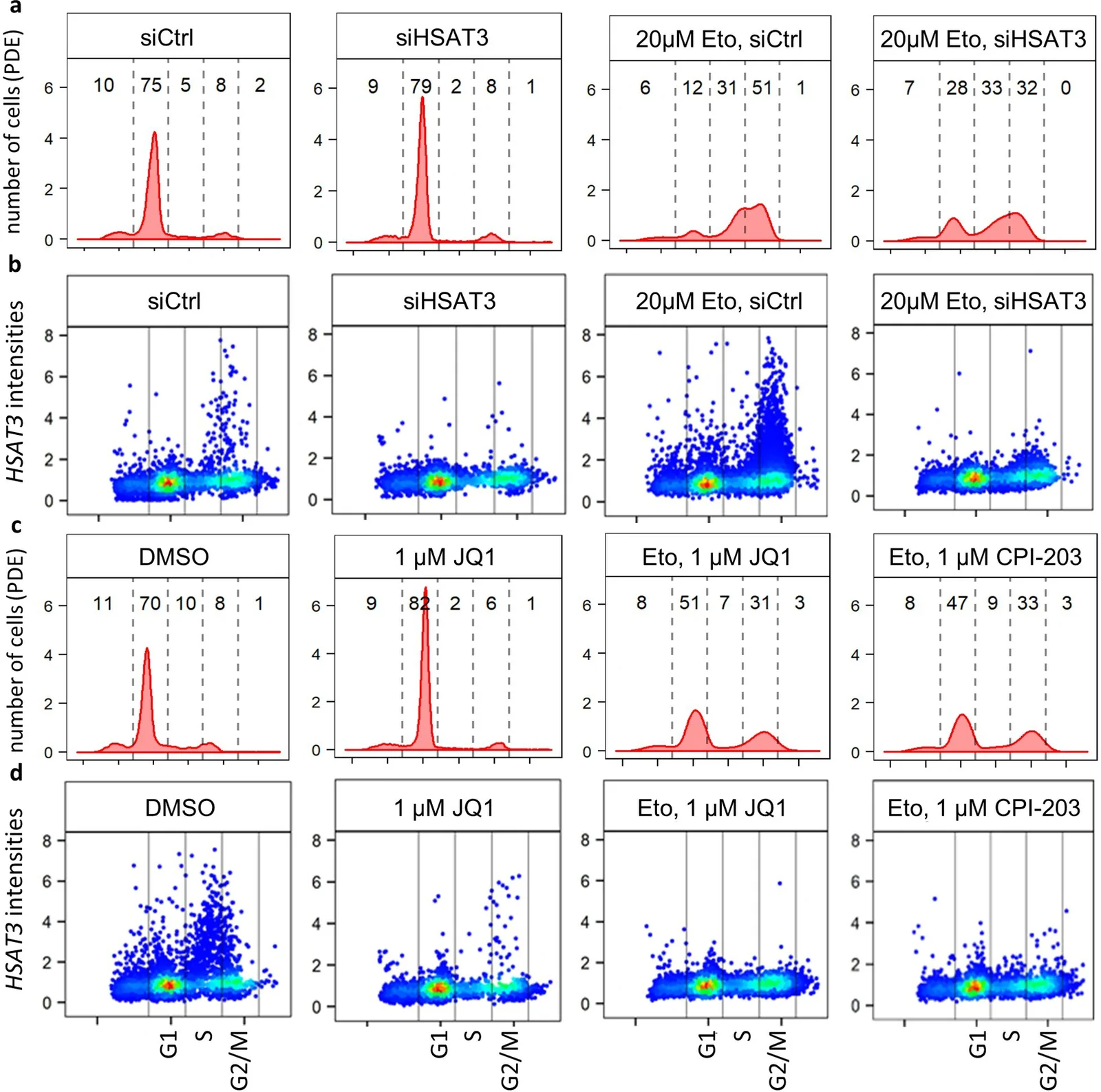
*HSAT3* knockdown alters cell cycle progression of etoposide treated cells. **a**, **c** Cell cycle distribution profiles and **b**, **d** *HSAT3* intensities of HeLa cells after thermal stress. Cells were subjected to different treatments as indicated in the figure, followed by heat shock for 1 h at 44 °C, subjected to *HSAT3* RNA-FISH and stained with Hoechst staining dye. Nuclei were counted with an automated high-content microscopy approach. **a**, **d** Cell cycle stages were determined by the intensity of the Hoechst staining and cell numbers were calculated by partial differential equation. **b**, **d** *HSAT3* signal intensity is given on the y-axis, the number of cells in the different cell cycle phase is color coded as density plot. **a**, **b** Cells were either subjected to siControl or *siHSAT3* treatment alone or in combination with 20 µM etoposide. **c**, **d** HeLa cells were either treated with DMSO, JQ1 (1 µM), a combination of etoposide (5 µM) and JQ1 (1 µM) or a combination of etoposide (5 µM) and CPI-203 (1 µM)

HeLa cells were transfected with control or *HSAT3*-targeting siRNA and 24 h after transfection, subjected to heat stress (1 h at 44 °C), followed by *HSAT3* RNA-FISH and quantitative imaging-based cell cycle analysis (Fig. 2, Additional Fig. 6). Heat stress alone resulted in the expected enrichment of cells in G1 phase, and this distribution was not markedly altered by *HSAT3* knockdown.

In contrast, treatment with the TOP2A inhibitor etoposide caused accumulation of cells in late S and early G2 phases [37], consistent with replication-associated checkpoint activation (Fig. 2a, Additional Fig. 6). We then tested combination treatments of etoposide and *HSAT3* knockdown. For combination treatments involving siRNA and a compound, the cells were treated with an siRNA, and then subjected to heat stress for 1 h at 44 °C, 24 h later. The indicated compound was applied directly after heat stress. At 5 µM etoposide, only minor changes were observed (Additional Fig. 6). However, at 20 µM etoposide, *HSAT3* depletion led to a reduction of cells in G2 phase and a concomitant increase in G1 phase compared to control siRNA-treated cells.

We next examined *HSAT3* induction across cell cycle phases. As expected, *HSAT3* foci were not detected under non-stress conditions. Following heat stress, *HSAT3* signals were strongly enriched in late S and early G2/M phases. This induction was further enhanced by combined heat stress and etoposide treatment and was efficiently suppressed by *HSAT3* knockdown (Fig. 2b).

Given that *HSAT3* expression is regulated by BET proteins, we assessed whether BET inhibition phenocopies *HSAT3* depletion. Cells were treated directly after a 1 h heat stress at 44 °C with the indicated compounds. Treatment with the BET inhibitors JQ1 or CPI-203 alone resulted in a predominance of cells in G1 phase, similar to DMSO controls. Importantly, combined treatment of etoposide with either inhibitor shifted the cell population toward G1 and reduced S/G2 fractions compared to etoposide alone (Fig. 2c, Additional Fig. 6). Consistently, both inhibitors markedly reduced *HSAT3* foci formation under heat stress conditions, either alone or in combination with etoposide (Fig. 2d).

### *HSAT3* depletion impairs replication fork restart

Given the association of *HSAT3* with TOP2A, a key regulator of DNA topology during replication, we next investigated whether *HSAT3* influences DNA replication dynamics.

**Fig. 3:**
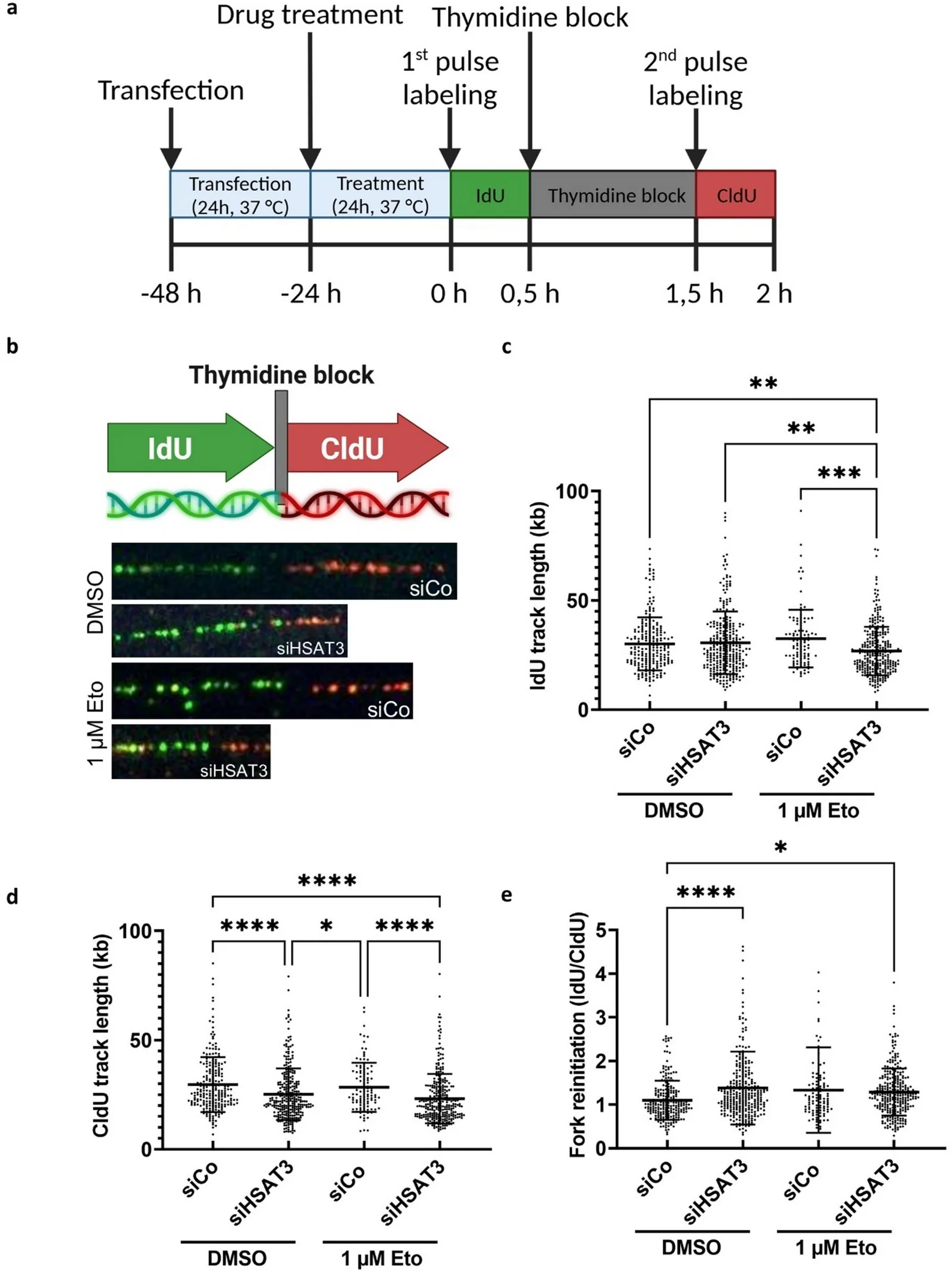
*HSAT3* knockdown affects replication re-initiation. **a** Workflow of the DNA fiber assay to analyze DNA replication progression and re-initiation. HeLa cells were transfected with a siRNA targeting *HSAT3* or a non-targeting siRNA. After 24 h, cells were treated with 1 µM etoposide or DMSO. 48 after transfection, cells were pulse labelled with IdU (green) for 30 min, stalled with thymidine (60 min) and labelled with CldU (red) for 30 min. **b** Representative IdU and CldU replication tracks **c**, **d** Track lengths of **c** IdU or **d** CldU labelled DNA fibers and **e** IdU to CldU track length ratio (Fork re-initation). Significance was tested using a two-way ANOVA followed by Tukey’s multiple comparison test. P-values are marked with *P* ≤ 0.0001 (****), *P* ≤ 0.01 (**), P *<* 0.05 (*). Only significant comparisons are shown

We performed DNA fiber assays in HeLa cells following *HSAT3* knockdown, with or without etoposide treatment. Replication was monitored by sequential incorporation of IdU and CldU to assess ongoing replication and replication restart, respectively (Fig. 3a, b). Efficient *HSAT3* depletion was confirmed by RT-qPCR (Additional Fig. 5).

*HSAT3* knockdown alone, as well as treatment with low-dose etoposide (1 µM), did not significantly affect ongoing replication, as indicated by IdU track length. However, combined *HSAT3* depletion and etoposide treatment resulted in a reduction of track length, suggesting impaired replication progression (Fig. 3c).

Strikingly, *HSAT3* knockdown significantly reduced replication re-initiation efficiency, as reflected by decreased CldU track length. This defect was further exacerbated upon combined treatment with etoposide, whereas etoposide alone had no significant impact on replication restart (Fig. 3d, e). These findings indicate that *HSAT3* is required for efficient replication re-initiation, particularly under replication stress conditions.

### Reduced *HSAT3* expression delays tumor growth in NSCLC xenograft models

To assess the functional relevance of *HSAT3* in tumor growth *in vivo*, we employed a cell line-derived xenograft (CDX) model using the lung cancer cell line HCC827 with stable *HSAT3* knockdown, that we had previously characterized and efficient depletion of *HSAT3* was demonstrated [19].

**Fig. 4:**
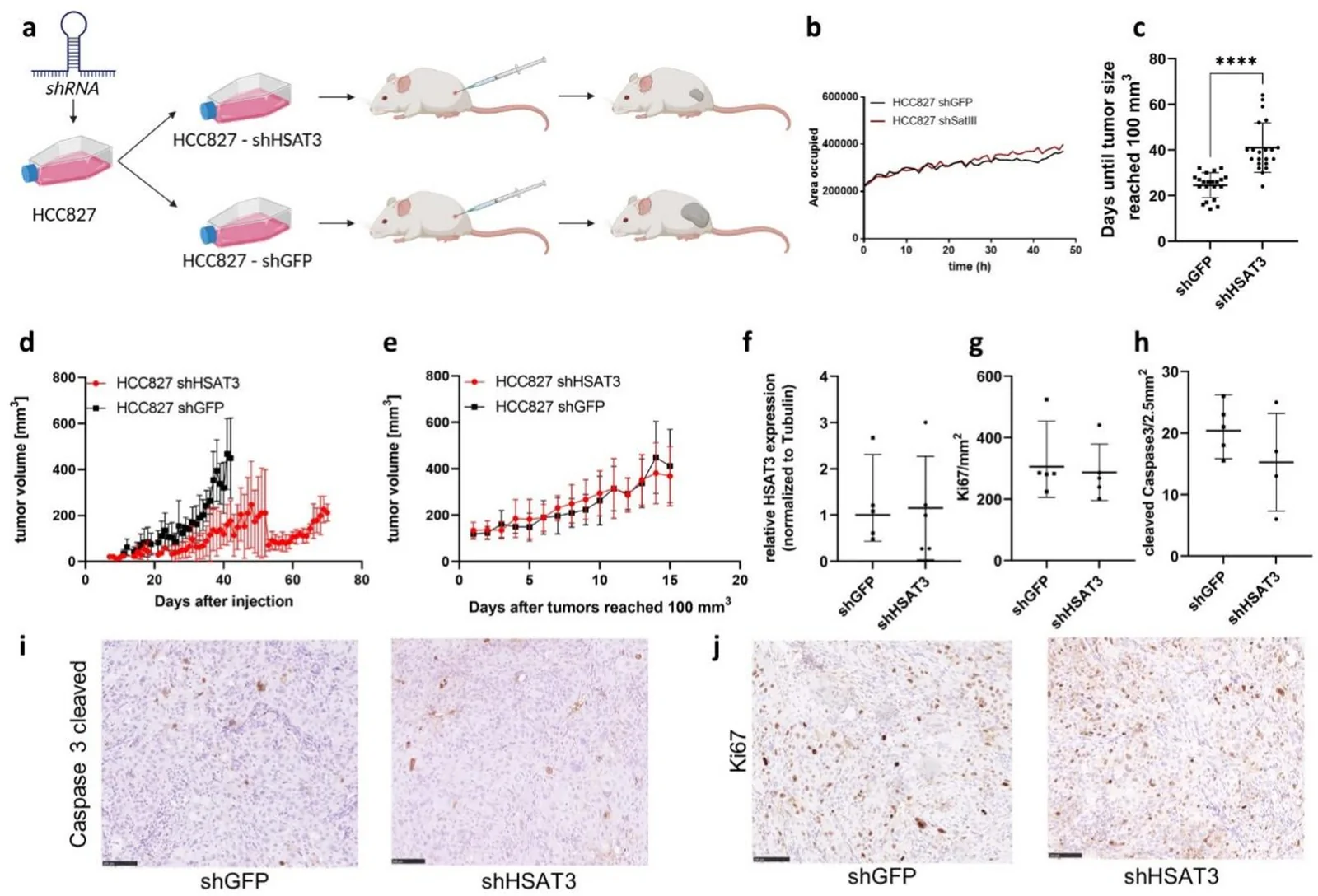
*HSAT3* knockdown delays tumor growth in CDXs. **a** Workflow of NSCLC-CDX establishment through injection of HCC827 cells stably expressing shRNA targeting *HSAT3* or GFP as a negative control. **b** Cell proliferation assay of the stable HCC827-shGFP and the HCC827-shHSAT3 cell lines **c** Days until tumors reached a volume of 100 mm^3^ in CDX injected with HCC827 cells stably expressing shRNA targeting *HSAT3* or *GFP*. Significance was calculated using a two-tailed Mann-Whitney test. **d** Tumor volume over time after injection. shHSAT3, *n* = 21, shGFP, *n* = 22. **e** Tumor volume after tumors had reached a volume of 100 mm^3^. shHSAT3, *n* = 8, shGFP, *n* = 9 (**d**, **e**) given are the mean +/-SD. **f** Quantitative RT-PCR showing the relative *HSAT3* expression in the xenografts tumors, two weeks after the tumors reached a volume of 100 mm^3^. **g** Number of Ki67 positive cells per mm2 in shGFP and shHSAT3 xenografts. **h** Number of cleaved Caspase 3 positive cells per 2.5 mm^2^ in shGFP and shHSAT3 xenografts. **i**, **j** Representative examples of the immunohistochemistry for **i** cleaved Caspase 3 and **j** Ki67

Immunodeficient mice were subcutaneously injected with either control (shGFP) or *HSAT3*-depleted (sh*HSAT3*) HCC827 cells, and tumor growth was monitored over time (Fig. 4a). It is worth noting that we observed no difference in growth when comparing HCC827-shGFP and HCC827-shHSAT3 cells in cell proliferation assays (Fig. 4b). While both groups formed tumors, *HSAT3* knockdown resulted in a marked delay in tumor progression compared to control tumors (Fig. 4c, d).

Quantitative analysis revealed a significant reduction in tumor growth kinetics in the *HSAT3*-depleted group, indicating that *HSAT3* expression contributes to efficient tumor expansion *in vivo*. This effect is consistent with our *in vitro* findings showing that *HSAT3* supports replication re-initiation and cell cycle progression under stress conditions. However, after the tumors reached a size of 100 mm^3^, the growth curves of *HSAT3*-depleted and control HCC827 cells were similar (Fig. 4e). Two weeks after tumor volume reached 100 mm^3^, we tested *HSAT3* expression by quantitative PCR and observed no difference between the two groups at this time point (Fig. 4f). Consistent with this observation, immunohistochemical analysis of five tumors per group revealed no significant differences in either Ki67 or cleaved caspase-3 staining (Fig. 4g–j).

Together, these data demonstrate that *HSAT3* promotes tumor growth in NSCLC models, likely by facilitating replication-associated processes and enhancing cellular adaptation to stress.

### Epigenetic compound screening identifies regulators of *HSAT3* expression and sensitizers to etoposide

**Fig. 5:**
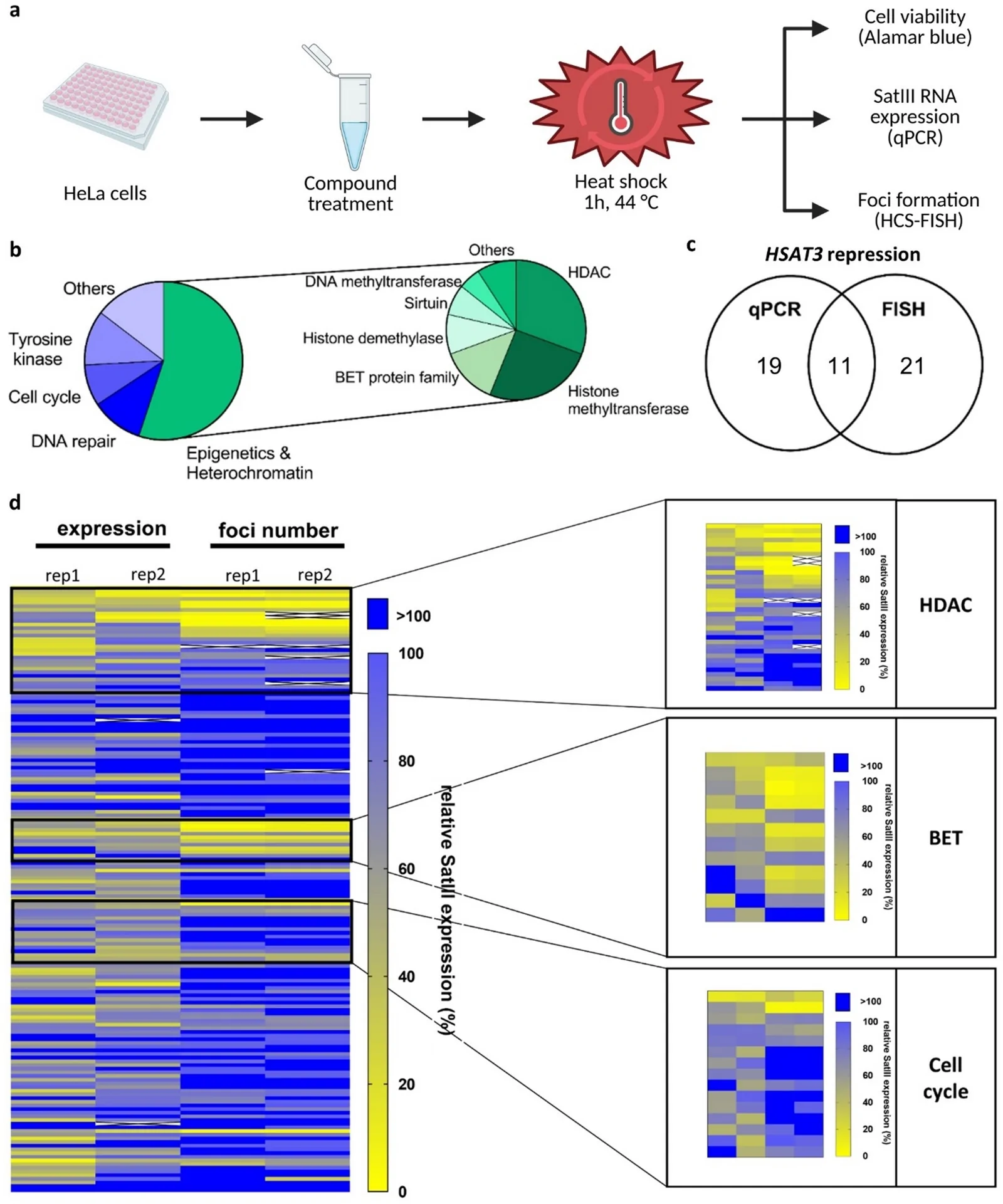
Compound screen reveals regulators of *HSAT3* expression and foci formation. **a** Schematic workflow of the compound screening. HeLa cells were seeded and after 24 h heat shocked for 1 h at 44 °C. After HS, the cells were treated with one of the 178 compounds to then perform HCS-FISH, RT-qPCR and a cell viability assay. **b** Composition of the epigenetic compound library. Relative fraction of functional subgroups, including a magnification of the subgroup ‘Epigenetics & Heterochromatin’. **c** Venn diagram of the results of the compound screen. Out of a total number of 178 compounds, 11 compounds showed both, a decrease of *HSAT3* expression and foci formation. **d** Results of the epigenetic compound screen. Heat map indicates expression of *HSAT3* for both read-out methods (% to DMSO as reference): RNA expression by qPCR (left columns) and *HSAT3* foci formation by HCS-FISH (right columns). Both replicates for each method are shown. RNA expression and foci numbers were normalized to solvent (DMSO)

To systematically identify pharmacological regulators of *HSAT3* expression, we performed a targeted screen using a curated library of 178 epigenetic compounds in HeLa cells. Cells were pre-treated with individual compounds followed by heat stress to induce *HSAT3* expression, and *HSAT3* levels were quantified by RT-qPCR and high-content RNA-FISH analysis (Fig. 5a, b, Additional Fig. 6).

The screen revealed a subset of compounds that suppressed *HSAT3* induction upon heat stress (*p* < 0.05, fold change < 0.666), (Fig. 5c). Among the most prominent hits were inhibitors targeting bromodomain and extraterminal domain (BET) proteins, consistent with the known role of BRD4 in *HSAT3* transcriptional regulation. In addition, compounds affecting chromatin modifiers and cell cycle regulators also reduced *HSAT3* levels, suggesting that *HSAT3* expression is broadly controlled by epigenetic pathways (Fig. 5d, Supplementary Table 2).

Quantitative imaging confirmed that these compounds not only reduced overall *HSAT3* transcript levels but also markedly decreased the formation of nuclear stress bodies. This indicates that pharmacological inhibition of *HSAT3* expression effectively disrupts stress-induced nuclear compartmentalization (Fig. 5d).

Since we previously observed increased sensitivity to etoposide following *HSAT3* knockdown using siHSAT3, ASO-HSAT3, and stable HCC-shSAT3 knock down cells [19], we evaluated whether *HSAT3*-suppressing compounds modulate cellular sensitivity to the TOP2A inhibitor etoposide. Notably, several HDAC inhibitors identified in the screen enhanced etoposide-induced cytotoxicity, indicating a synergistic interaction (Additional Fig. 9).

Together, these results identify epigenetic regulators of *HSAT3* expression and suggest that pharmacological suppression of *HSAT3* represents a potential strategy to enhance the efficacy of TOP2A-targeting therapies.

## Discussion

In this study, we investigated the role of the stress-induced pericentromeric lncRNA *HSAT3* in nuclear stress body formation, DNA replication, and chemotherapeutic response. Our data support a model in which *HSAT3* contributes to the spatial organization of stress-responsive nuclear compartments and influences replication-associated processes, cell cycle behavior, and sensitivity to etoposide.

A central finding of our study is that TOP2A is recruited to *HSAT3*-containing nuclear stress bodies during recovery from thermal stress and can be precipitated by *HSAT3* RNA oligonucleotides. Binding of TOP2A to RNA has been previously reported [38, 39] These observations extend our previous work showing that aberrant *HSAT3* expression contributes to etoposide resistance [19]. Nuclear stress bodies are known to arise in response to thermal stress at pericentromeric satellite loci, most prominently at 9q12, and to function as dynamic membrane less compartments that recruit RNA-binding proteins and regulatory factors [9, 11–13, 20, 34, 40]. Previous work has shown that *HSAT3*-containing nuclear stress bodies promote stress-induced intron retention and coordinate post-transcriptional regulatory events through factors such as CLK1 and YTHDC1 [3, 15, 16]. In addition, BRD4 has been implicated in the regulation of stress-induced splicing responses [18]. Our results now suggest that the spectrum of *HSAT3*-associated proteins extends beyond splicing-associated factors and may also include proteins with direct roles in DNA topology and replication-associated chromatin transactions.

This interpretation is consistent with the broader concept that repetitive non-coding RNAs are not merely transcriptional by-products of heterochromatin relaxation, but can function as structural and regulatory molecules within the nucleus. Pericentromeric and satellite-derived transcripts have previously been linked to chromosomal stability, heterochromatin organization, RNA hybrid formation, activation of DNA damage signaling, and defective mitotic progression when aberrantly expressed [1, 3–5, 9–25, 27–29]. In this context, the recruitment of TOP2A to *HSAT3*-positive nuclear stress bodies may reflect a stress-adaptive mechanism that coordinates local chromatin organization and genome maintenance during recovery. At the same time, it is conceivable that excessive or prolonged *HSAT3* accumulation redistributes TOP2A in a way that affects its availability at other genomic sites, which would be consistent with our earlier findings on etoposide response [19].

Our DNA fiber analyses further indicate that *HSAT3* depletion delays replication re-initiation. This result is particularly interesting in light of prior reports showing that satellite transcripts can affect replication fidelity and induce replication stress [26, 28]. Overexpression of murine major satellite RNAs has been shown to impair replication fidelity and to generate immediate replication-associated defects [26]. In parallel, faithful replication of pericentromeric regions themselves has been identified as an important prerequisite for genome stability, and proteins such as TRF2 and RTEL1 have been shown to support replication through these repetitive domains [28, 30]. Moreover, heterochromatin perturbation and satellite derepression have been closely associated with replication stress, genome instability, and RNA hybrid formation [8, 23–29]. Against this background, our data suggest that *HSAT3* may contribute to the efficient restart or re-initiation of DNA replication following transient perturbation. Mechanistically, several non-mutually exclusive possibilities can be considered. First, *HSAT3* may influence the availability or subnuclear distribution of TOP2A during stress recovery. Second, *HSAT3* may alter the organization of pericentromeric chromatin in a manner that affects replication restart. Third, *HSAT3*-dependent nuclear stress body formation could indirectly regulate replication through stress-responsive RNA processing pathways, given the established links between nuclear stress bodies, BRD4, CLK1, YTHDC1, and heat-shock-induced splicing regulation [3, 14–16, 18, 19].

At present, our data do not distinguish whether the delayed re-initiation phenotype primarily reflects altered origin firing, impaired fork restart, reduced fork progression, or a broader defect in replication-associated stress recovery. Nevertheless, the phenotype is compatible with the idea that satellite-derived RNAs participate in coordinating nuclear functions that become particularly relevant in repetitive and heterochromatin-rich genomic regions. This fits with previous evidence that heterochromatin perturbation and derepression of satellite transcription are closely associated with replication stress, genome instability, and RNA hybrid accumulation [24–29]. Because pericentromeric regions are intrinsically difficult to replicate, changes in *HSAT3* abundance could have disproportionately strong consequences for the successful re-establishment of replication after stress.

Our cell cycle analyses further support a role for *HSAT3* in stress adaptation. Under heat stress alone, *HSAT3*-positive cells were enriched in late S phase and early G2/M, consistent with our previous observations and with the notion that nuclear stress bodies are particularly prominent in cells progressing through these phases [19]. In the presence of etoposide, *HSAT3* knockdown altered cell cycle distribution and shifted cells away from G2-phase accumulation. These findings again align with our earlier work showing that elevated *HSAT3* levels promote resistance to the TOP2A inhibitor etoposide [19]. Together, the current and previous data support a model in which *HSAT3* contributes to cellular adaptation to genotoxic and proteotoxic stress by modulating the localization and possibly the function of factors required for DNA topology control, RNA processing, and cell cycle progression.

The compound screen adds an important translational dimension to this model. Because *HSAT3* expression is controlled by HSF1 and BET family members, especially BRD4 [17–20], and because BRD4 also regulates heat-shock-associated splicing responses [18], we reasoned that pharmacological interference with chromatin-associated regulators might modulate *HSAT3* levels. Our screen indeed identified compounds that suppress *HSAT3* expression and/or nuclear stress body formation, including inhibitors targeting epigenetic regulators. This finding is conceptually consistent with the established role of BET proteins in *HSAT3* induction and nuclear stress body assembly, as pharmacological BET inhibition by JQ1 or I-BET151 has previously been shown to block *HSAT3* foci formation while leaving HSF1 foci largely unaffected [18, 19]. In addition, perturbations of chromatin states, including loss of BRCA1-dependent H2A ubiquitination, reduced H3K9 methylation, or altered histone acetylation such as SIRT6-dependent control of H3K18 acetylation, have been linked to increased satellite transcription and genome instability [24–29]. The present screening results therefore support the idea that *HSAT3* is pharmacologically tractable and that its expression depends on a broader chromatin-regulatory network.

Importantly, compounds identified in the screen also increased sensitivity to etoposide in proliferation assays, in agreement with our previous findings that *HSAT3* depletion re-sensitizes cells to TOP2A inhibition [19]. While these data do not yet establish that all compound effects are mediated specifically through *HSAT3*, they support the principle that repression of *HSAT3* can be exploited in combination-treatment settings. This may be particularly relevant in tumors with elevated *HSAT3* expression, activated heat-shock signaling, or chromatin states permissive for satellite derepression [21–23]. Thus, the screen not only identifies candidate regulators of *HSAT3* biology, but also provides a first framework for pharmacological targeting of stress-induced repetitive RNAs in cancer.

Our xenograft data are consistent with this idea, as stable *HSAT3* knockdown delayed tumor growth in vivo. Together with the cell culture findings, this suggests that *HSAT3* contributes not only to acute stress responses but also to longer-term tumor cell fitness. Given the established links between satellite derepression, chromatin dysfunction, and genome instability [24–29], it is conceivable that *HSAT3* supports tumor growth by helping cancer cells tolerate replication-associated and transcription-associated stress. Future studies will be needed to determine whether this effect is primarily mediated through TOP2A-related functions, stress-body-dependent RNA regulation, altered heterochromatin dynamics, or a combination of these mechanisms.

Several limitations of the present study should be considered. While our data support an association between *HSAT3* and TOP2A, the precise molecular nature of this interaction and its impact on TOP2A enzymatic activity remain to be defined. In addition, although DNA fiber analyses indicate delayed replication re-initiation upon *HSAT3* depletion, further studies will be required to dissect the relative contributions of origin firing, fork progression, and replication stress signaling.

Finally, while our compound screen identifies candidate regulators of *HSAT3* expression, additional work will be needed to determine the specificity and mechanistic basis of these effects.

Despite these limitations, our study expands the functional landscape of *HSAT3* beyond its established role in stress-dependent RNA processing. By linking *HSAT3* to TOP2A, replication re-initiation, cell cycle behavior, tumor growth, and pharmacological response, our findings support the emerging view that repetitive RNAs actively participate in the organization of nuclear processes under stress conditions. More broadly, they highlight stress-induced satellite RNAs as potentially actionable regulators at the interface of chromatin organization, genome maintenance, and cancer therapy.

## Conclusion

Together, our findings establish a functional link between stress-induced repetitive RNAs, nuclear compartmentalization, and replication-associated processes. By demonstrating that *HSAT3* influences TOP2A localization, replication re-initiation, and cellular responses to chemotherapeutic stress, this study expands the current understanding of how pericentromeric lncRNAs contribute to genome maintenance.

More broadly, our results support a model in which stress-induced satellite RNAs actively participate in the coordination of nuclear processes under adverse conditions and highlight *HSAT3* as a potential target for therapeutic intervention in cancer.

## Supplementary Information

Below is the link to the electronic supplementary material.

Additional file 1. Additional Fig. 1: TOP2A is recruited to nSB in HeLa cells. RNA-FISH for *HSAT3* and immunofluorescence for TOP2A were performed in HeLa, nuclei were stained with Hoechst Dye. Images are shown for the indicated timepoints without thermal stress (nHS) and after thermal stress for 1 hour at 44°C, followed by 0–24 hours of recovery at 37°C. The white boxes refer to cells shown in Fig. 1.

Additional file 2. Additional Fig. 2: TOP2A is recruited to nSB in HeLa cells. RNA-FISH for *HSAT3 *and Immunofluorescence for TOP2A were performed in HeLa, nuclei were stained with Hoechst Dye. Images are shown for the indicated timepoints without thermal stress (nHS) and after thermal stress for 1 hour at 44°C, followed by 0–24 hours of recovery at 37°C. The white boxes refer to cells shown in Fig. 1.

Additional file 3. Additional Fig. 3: Quantification of *HSAT3 *and TOP2A foci. A) Quantification of *HSAT3 *and TOP2A foci and of foci with *HSAT3* and TOP2A colocalization. Given are the averages of three images. B) Manders’ coefficients (M1 and M2). M1 gives the co-occurrence fraction of the *HSAT3 *with the TOP2A signal and M2 the co-occurrence fraction of the TOP2A with the *HSAT3 *signal.

Additional file 4. Additional Fig. 4: TOP2A is recruited to nSB in the NSCLC cell line A549. RNA-FISH for *HSAT3 *and Immunofluorescence for TOP2A were performed in A549, nuclei were stained with Hoechst Dye. Images are shown for the indicated timepoints without thermal stress (nHS) and after thermal stress for 1 hour at 44°C, followed by 0–24 hours of recovery at 37°C.

Additional file 5. Additional Fig. 5: TOP2A binds to a *HSAT3* oligonucleotide in the NSCLC cell lines HCC827 and A549. In vitro pulldown assays of RNA-protein (RNP) complexes with *HSAT3*. A biotinylated *HSAT3*-RNA oligo (GGAAU)x4, a negative control oligo (CCCCCAAAAAUUUUUGGGGG) were incubated with HCC827 or A549 cell extracts. Cells were kept at 37°C (nHS) or exposed to thermal stress for 1 h at 44°C (HS). 7% of the Input (63 µg) was loaded on the SDS page. TOP2A and ALYREF were detected by Western blotting.

Additional file 6. Additional Fig. 6: Additional cell cycle profiles. Cell cycle profiles of heat shocked HeLa cells treated with 5 µM etoposide and a non-targeting siRNA or an siRNA targeting *HSAT3* as well as single treatment with the BET inhibitor CPI-203 (1µM).

Additional file 7. Additional Fig. 7: *HSAT3* expression in cells used for the replication studies. HeLa cells were either transfected with an siRNA targeting *HSAT3* or a non-targeting control and treated with etoposide (1µM) or DMSO. Expression was analyzed by qPCR. Error bars represent SD of the mean of n = 3 technical replicates. P-values < 0.01 are marked with (**); p < 0,05 with (*). Significance was determined using two-tailed unpaired Student’s t-test.

Additional file 8. Additional Fig. 8: Cell numbers of the compound screen. Cells were analysed by High-Content-Microscopy Screening after treatment with the indicated compounds. Shown is the mean of two individual replicates.

Additional file 9. Additional Fig. 9. Combination treatment of etoposide and HDAC inhibitors. Combination treatments of compounds reducing *HSAT3* expression with etoposide. Cell viabilities of HeLa cells treated with increasing etoposide concentrations alone and in combination with compounds identified in the screen. Directly after exposure to 1 h HS at 44°C, cells were treated with the indicated etoposide concentrations alone or in combination with one concentration of the indicated compound. After an additional 48 h, cell viabilities were measured using AlamarBlue®. (a) 0.1 µM of the HDAC inhibitor belinostat. (b) 0.1 µM of the HDAC inhibitor trichostatin A (TSA). (c) 1 µM of the HDAC inhibitor M344. Cell viabilities were normalized to the DMSO control for single treatments and to the corresponding compound for double treatments.

Additional file 10. Additional Table 1: Oligonucleotide sequences.

Additional file 11. Additional Table 2: Compound library.

## Data Availability

The datasets used during the current study are available from the corresponding author on reasonable request.
