# Supplementary material for: HSAT3 lncRNA regulates replication re-initiation and colocalizes with TOP2A under stress conditions
Source: Epigenetics Chromatin. 2026 Oct 24;19:46. doi: 10.1186/s13072-026-00707-9 (PMC13700243; doi:10.1186/s13072-026-00707-9)
Supplement: Supplementary file 1 [file 13072_2026_707_MOESM3_ESM.pdf]

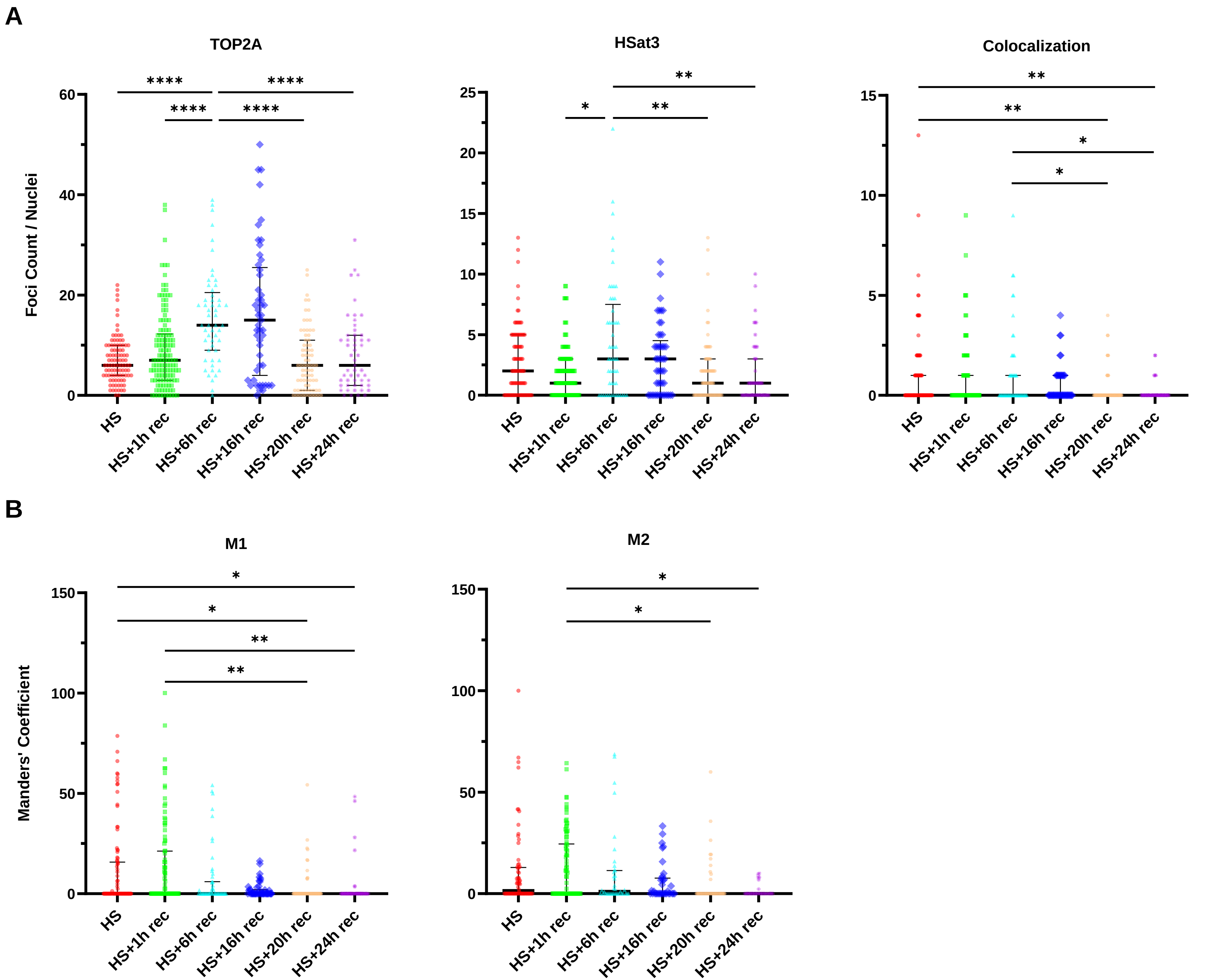
A
TOP2A
HSat3
Colocalization
Foci Count / Nuclei
0
20
40
60
0
5
10
15
20
25
0
5
10
15
****
****
****
****
*
**
**
**
**
*
*
HS
HS+1h rec
HS+6h rec
HS+16h rec
HS+20h rec
HS+24h rec
B
M1
M2
Manders' Coefficient
0
50
100
150
*
*
**
**
*
*
