# Supplementary material for: HSAT3 lncRNA regulates replication re-initiation and colocalizes with TOP2A under stress conditions
Source: Epigenetics Chromatin. 2026 Oct 24;19:46. doi: 10.1186/s13072-026-00707-9 (PMC13700243; doi:10.1186/s13072-026-00707-9)
Supplement: Supplementary file 2 [file 13072_2026_707_MOESM10_ESM.docx]

| **Supplementary Table 1: Oligonucleotides used in this study.** | | | |  |
| --- | --- | --- | --- | --- |
|  |  |  |  |  |
| **Experiment** | **Gene** | **Sequence name** | **Sequence (5’-3’)** | **Source** |
| Reverse transcription | HSAT3 | RSM13 | ccgtaaaacgacggccagttcccttccattccattattatcc | Valgardsdottir et al., 2007 |
|  |  | FSM13 | ccgtaaaacgacggccagtcagtggaaggcattagaatcaac |  |
|  | Random | Random Hexameres | Random Hexameres | Metabion |
|  |  |  |  |  |
| qPCR | HSAT3-RNA | Hur98-R | aatcaacccgagtgcaatcg | Valgardsdottir et al. 2007 |
|  |  | M13 | ccgtaaaacgacggccag |  |
|  | TUBB | TUBB-fw | gctggaccgcatctctgtgta | Kanne et al., 2019 |
|  |  | TUBB-rv | agagtccatggtcccaggtt |  |
|  |  |  |  |  |
| Gene knockdown | HSAT3 | siHSAT3 | uggaauggaauggaauggadtdt | Kanne et al., 2019 |
|  |  |  | uccauuccauuccauuccadtdt |  |
|  |  | shHSAT3 | ccggtggaatggaatggaatggaaggatccatccattccat | Kanne et al., 2019 |
|  |  |  | tccattccatttttg |  |
|  | Ctrl | siCo | #D-001810-10-50, On-Target Plus Non-targeting Control Pool, | Dharmacon, Horizon |
|  | GFP | shGFP | ccgggcaagctgaccctgaagttcaggatccagaacttcagggtcagcttgctttttg | Kanne et al., 2019 |
|  |  |  | aattcaaaaagcaagctgaccctgaagttctggatcctgaacttcagggtcagcttgc |  |
| In vitro pulldown assay | HSAT3 | SatIII | ggaauggaauggaauggaau-Biotin-TEG | This Paper |
|  | Ctrl | Ctrl | Biotin-cccccaaaaauuuuuggggg | This Paper |
| HSAT3 FISH probes | HSAT3 | HSAT3_G-rich_1 | cattccattccattccattc | Kanne et al., 2019 |
|  |  | HSAT3_G-rich_2 | cattccattgcattccattc |  |
|  |  | HSAT3_G-rich_3 | tgattccattccattccatt |  |
|  |  | HSAT3_G-rich_4 | attgcattccattccattcc |  |
